# Lipoidal Soft Hybrid Biocarriers of Supramolecular Construction for Drug Delivery

**DOI:** 10.5402/2012/474830

**Published:** 2012-07-19

**Authors:** Dinesh Kumar, Deepak Sharma, Gurmeet Singh, Mankaran Singh, Mahendra Singh Rathore

**Affiliations:** Department of Pharmaceutics, CT Institute of Pharmaceutical Sciences, Jalandhar 144020, India

## Abstract

Lipid-based innovations have achieved new heights during the last few years as an essential component of drug development. The current challenge of drug delivery is liberation of drug agents at the right time in a safe and reproducible manner to a specific target site. A number of novel drug delivery systems has emerged encompassing various routes of administration, to achieve controlled and targeted drug delivery. Microparticulate lipoidal vesicular system represents a unique technology platform suitable for the oral and systemic administration of a wide variety of molecules with important therapeutic biological activities, including drugs, genes, and vaccine antigens. The success of liposomes as drug carriers has been reflected in a number of liposome-based formulations, which are commercially available or are currently undergoing clinical trials. Also, novel lipid carrier-mediated vesicular systems are originated. This paper has focused on the lipid-based supramolecular vesicular carriers that are used in various drug delivery and drug targeting systems.

## 1. Introduction

The turn of century has witnessed a remarkable growth in drug discovery, development, and use. New drug delivery technologies are revolutionizing the development and creating R&D-focused pharmaceutical industries to suit the needs of the modern world. The scenario of pharmaceutical research is being steadily changed, by encouraging development of novel drug delivery of existing drug molecule instead of development of new chemical entities. The novel drug delivery approaches aim to develop a carrier system which can hold the molecule effectively and then navigate them towards the right destination without affecting the physiological conditions of the body. Vesicular system has achieved new heights during the last few years as an essential component of drug development. The phospholipid-mediated drug delivery has emerged as a powerful methodology for the treatment of various pathologies. The therapeutic index of traditional and novel drugs is enhanced via the increase of specificity due to targeting of drugs to a particular tissue, cell or intracellular compartment, the control over release kinetics, the protection of the active agent or a combination of the above [1–3]. From the last two decades, microparticulate lipoidal vesicular systems have been under extensive investigation as carriers for the improved delivery of a broad spectrum of agents, including chemotherapeutic agents, imaging agents, antigens, immunomodulators, chelating compounds, haemoglobin and cofactors, lipids, and genetic material [4].

Phospholipids are major components of plasma membrane and organelle membranes that maintain the integrity of the cell or organelles by creating a semi-impermeable barrier from their outside environment. In normal cells, phospholipids are asymmetrically distributed in inner and outer leaflets of plasma membrane, with phosphatidylcholine (PC) and sphingomyelin (SM) predominantly in the outside leaflet and phosphatidylserine (PS) and phosphatidylethanolamine (PE) in the inner leaflet of plasma membrane [5]. These constitute basic skeleton of supra molecular construction. A special amphiphilic feature of phospholipids makes them suitable to be used as excipients for poorly water-soluble drugs. They get hydrated, form micelles or are organized as lipid bilayers with the hydrophobic tails lined up against one another and the hydrophilic head-group facing the water on both sides [6].

Various such systems, which have gained an utmost importance, like vesicular systems and microparticulate systems including liposomes, niosomes, pharmacosomes, transfersomes, and sphingosomes [7–10].

## 2. Supramolecular System

**Figure 1:**
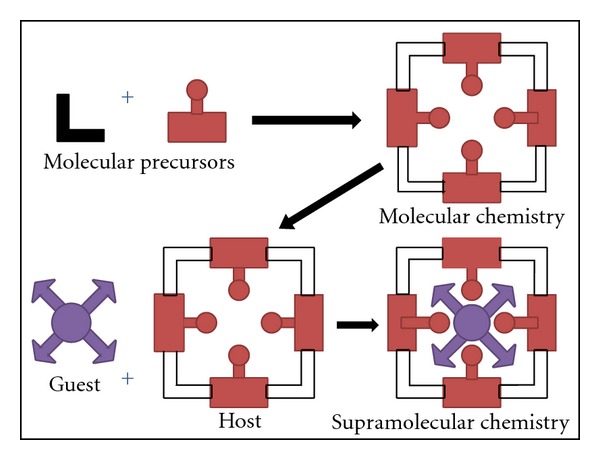
Comparison between the scope of molecular and supramolecular chemistry according to Lehn.

**Figure 2:**
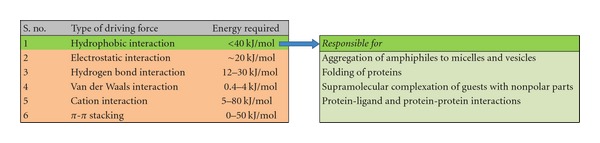
Driving forces for the formation of supramolecular structures and hydrophobic effects.

Supramolecular system is self-assembled, noncovalently bonded entity where complete molecular units are brought together through noncovalent forces to create a complex structure (as shown in Figure 1) [11]. The study of system involving aggregates of molecules or ions held together by noncovalent interactions, such as electrostatic interactions, dispersion interactions and solvophobic effects, known as supramolecular chemistry. The emergence of supramolecular chemistry has had a profound effect on how efficiently chemists prepare structures of different sizes and shapes using spontaneous secondary interactions such as hydrogen bonding, dipole-dipole, charge transfer, van der Waals, and *π*-*π* stacking interactions [12]. The energy requirement varies with type of driving force (as shown in Figure 2). The lipid bilayer structure, the fundamental structural unit of liposomes and vesicles, is a supramolecular assembly based on fuzzy interactions. Hydrophobic interactions are responsible for aggregation of amphiphiles to micelles and vesicles. Ambient temperature and amphiphilicity are two important factors for the formation and stability of vesicular structure. The lipid bilayer shows phase transition behavior depending on the ambient temperature. Therefore, the lipid bilayer behaves as a thermotropic liquid crystal. The driving force for the formation of the lipid bilayer structure is the amphiphilicity of the component molecules: one part of the molecule is soluble in a particular solvent while the other has a low affinity to the solvent. Formation of a bilayer structure is therefore driven by the self-assembling behavior of amphiphilic molecules or molecular complexes. Many types of amphiphile can form these supramolecular structures using the concepts of supramolecular chemistry. In this paper, we have tried to provide a topical overview and introduction to current thinking in supramolecular constructed vesicular systems and to show how supramolecular concepts evolving into pharmaceutical and therapeutic systems [13].

## 3. Vesicular System

Vesicles are typically made from lamellar liquid crystalline dispersions of lipids such as phosphatidylcholines, phosphatidylglycerols, and cholesterol, by various mechanical and/or chemical methods that act to disrupt the regular smectic stacking of the bilayers to produce separated bilayer sheets. Vesicles can be formed from a diverse range of amphiphilic building blocks. Biologic origin of these vesicles was first reported in 1965 by Bingham and was given the name Bingham bodies. These carrier systems can be modified or engineered accordingly, to slowly degrade, react to stimuli and be site-specific. Lipid vesicles are one type of many experimental models of biomembranes which evolved successfully, as vehicles for controlled delivery. Vesicular system has equal potential for the treatment of intracellular as well as extracellular infections. Vesicular drug delivery system has some of the advantages like:

- it is suitable for incorporation of both hydrophilic and lipophilic drugs,
- lipoidal covering improves the bioavailability especially in the case of poorly soluble drugs,
- it delays elimination of rapidly metabolizable drugs and thus functions as sustained release systems,
- it increased stability of bioactive via encapsulation,
- it increased efficacy and therapeutic index of drug [14].

At present, no available drug delivery system behaves ideally achieving all the lofty goals, but sincere attempts have been made to achieve them through novel approaches in drug delivery. A number of novel lipid carrier-mediated vesicular systems including cochleates [15], virosomes [16], emulsomes [17], archaeosomes [18], and bilosomes [19] have emerged encompassing various routes of administration.

## 4. Lipoidal Biocarriers 

Lipid-based formulations can be applied to influence the absorption of active ingredients via various mechanisms, such as modifying the release of active ingredients, improving their bioavailability, changing the composition and hence the character of the intestinal environment, stimulating the lymphatic transport of active ingredients, interacting with enterocyte-based transport processes, and reducing unwanted drug side effects. Phospholipids offer a number of opportunities to formulate DDS with drugs [6].

Liposomes have been extensively investigated as a potential drug delivery system due to the enormous diversity of structure and composition that can be achieved. They are considered as first generation vesicles. At present, there are so many existing lipid-based drug delivery technologies that having diversified methods of preparation and applications. However, an attempt is made to compile. Some of them are highlighted with unique features and therapeutic indications in Table 1. Proforms of some lipoidal carriers are summarized in Table 2.

### 4.1. Phytosomes

Phytosomes, often known as herbosomes, are recently introduced as an advanced dosage formulation technology to deliver herbal products and drugs by improved absorption and, as a result, produce better results than those obtained by conventional herbal extracts. Also, phytosomes exhibit better pharmacokinetic and pharmacodynamic profile than conventional botanical extracts. Phytosome is a patented technology developed by a leading manufacturer of drugs and nutraceuticals, to incorporate standardized plant extracts or water soluble phytoconstituents into phospholipids to produce lipid compatible molecular complexes, called as phytosomes and so vastly improve their absorption and bioavailability [43]. These are novel complexes, prepared by reacting phospholipid with phytoconstituents in ratio of 1 : 1 [44]. Polar, water soluble phytoconstituents like flavonoids, tannins, and glycosidic aglycones [45] experience better bioavailability, when enclosed in these vesicles, and ultimately dose requirement is reduced. Chemical bonding occurs between phytoconstituent and phosphatidylcholine molecule presents better stability of the complex. Phospholipids in phytosome possess hepatoprotective property as well as nutritional value providing energy for metabolism [46].

The fundamental difference between liposomes and phytosomes is that in liposomes the active principle is dissolved in the medium contained in the cavity or in the layers of the membrane, whereas in the phytosome it is an integral part of the membrane, being the molecules anchored through chemical bonds to the polar head of the phospholipid [53]. 

Complex of drug with phosphatidylcholine as a phytosome provides significant liver protection and enhanced bioavailability over conventional silymarin when taken orally. Phytosomal silybin results in faster absorbed formulation, as it absorbed at least four times more completely than silymarin, and ultimately dose was reduced [54]. Other benefits were also underlined like improvement in memory and brain functions,promotion ofadaptogenicfunctions,strengthening heart and cardiovascular systems [55].

### 4.2. Cochleates

Cochleates are cigar-like microstructures that consist of a series of lipid bilayer sheets rolled up in a spiral structure, formed as a result of the condensation of small unilamellar negatively charged liposomes [56]. Hydrophobic surface of sheets minimizes interaction with water. Cochleate technology is versatile for delivery of wide range of bioactives. It was shown to be effective in the therapeutic oral delivery of the hydrophobic drugs. These consists of soy-based phospholipids which can be phosphatidylserine (PS), dioleoylphosphatidylserine (DOPS), phosphatidic acid (PA), phosphatidylinositol (PI), and/or a mixture of one or more of these lipids with other lipids. Additionally, the lipid can include diphosphatidylglycerol (DPG), dioleoyl phosphatidic acid (DOPA), distearoyl phosphatidylserine (DSPS), dimyristoyl phosphatidylserine (DMPS), and dipalmitoyl phosphatidylglycerol (DPPG). A multivalent cation can be Zn^+2^ or Ca^+2^ or Mg^+2^ or Ba^+2^ and a drug can be protein,peptide,polynucleotide, anesthesic, steroidal anti-inflammatory agent, nutritional supplement, and/or herbal product [57–59].

Cochleates resist environmental attack, and their solid layered structure provides protection from degradation for the “encochleated” molecules, even when exposed to harsh environmental conditions or enzymes, including protection from degradation in the gastrointestinal tract, which makes them ideal candidates for drug delivery [15]. Cochleates can be prepared by any of the following methods:

- hydrogel method,
- trapping method,
- liposomes before cochleates (LC) dialysis method,
- direct calcium (DC) dialysis method,
- binary aqueous-aqueous emulsion system [57].

Cochleates are distinguished from liposomes by endowing characters like:

- water-free interior,
- less susceptible to oxidation,
- rod shape,
- rigid structure [58].

### 4.3. Carbohydrosomes

Carbohydrosomes are novel vesicular 3-dimensional structures formed from zwitterionic, cationic, or anionic carbohydrate-based lipids. These molecules self-assemble into liposome-like structures in an aqueous solution. These supramolecular structures are called carbohydrosome because these are carbohydrate analogues of glycerol-based liposomes. In carbohydrosome, glycerol back bone is replaced by ribose. Chemically carbohydrosome is Methyl-2, 3-di-o-lauroyl-*β*-D-ribose-5 phosphocholine (DLRPC). Alteration of conventional glycerol backbone by complete substitution provides new opportunity for assessing supramolecular structure formation and attaching macromolecules or ligands for biological targeting. The phase transition temperature (Tm) is 16°C higher than conventional liposome (DLPC). This increase in Tm indicates more efficient packing of bilayer below the Tm. According to Grrinstaff, carbohydrosomes are superior than glycerol-based liposomes because they possess (i) a larger backbone that increases the spacing between head and tail, (ii) an increase in hydrophobicity, and (iii) a decrease in backbone flexibility. Current modifications for conventional liposomes are limited to the hydrophobic tails and hydrophilic head groups but the carbohydrosomes can be modified at the ribose backbone also. This supramolecular structure forms a more stable liquid-crystallise phase below a transition temperature so it can be used as efficient delivery system through phase transition [3]. 

### 4.4. Archaeosomes

There has been growing interest of using liposomes prepared with lipid from lower microbes which can elicit strong cell-mediated and humoral immune responses against encapsulated antigen. Most of the microorganisms (including *Halobacterium salinarum*, *Methanobrevibacter smithii, Halococcus morrhuae*, and *Halorubrum tebenquichense*) possess glycerophospholipids with ester bond in their plasma membrane [60–63].

Archaeosomes are modified liposomes fabricated to comprise the unique glycerolipids of the nonpathogenic microbes. These lipids possess a core structures of archaeal polar lipids consisting of archaeol or diether lipid (2,3-di-O-diphytanyl-sn-glycerol) which contain 20 carbons per isoprenoid chain, while thermoacidophilic and some methanogenic archaea [64] synthesize caldarchaeol or tetraether lipid (2,2′-3,3′-tetra-O-dibiphytanyl-sn-diglycerol) containing 40 carbons per isoprenoid chain, and modifications of these structures. Basically, it is an ether-linked isoprenoid phytanyl core which engenders membrane stability, thereby promoting potent immune memory [65]. Additionally, the variable head domains of these glycerolipids have potent and unique APC-stimulating properties [66]. The distinct chemical structures of archaeal lipids confer considerable stability to the formed vesicular structures (archaeosomes) developed by using total polar lipids (TPL) of various archaebacteria [67]. Archaeosomes have been proven to be superior adjuvants, for evoking CD8^+^ T-cell responses [68], capable of facilitating strong as well as long lasting antigen delivery to the appropriate antigen processing compartment [69] and inducing strong humoral, cell-mediated, and memory responses [70]. They increase the nanovector stability [71] and promote immunostimulation [72]. 

Preparation of archaeosomes is similar to liposomes in that the archaeal lipids are extracted using chloroform/methanol/water from frozen thaw. Total polar lipids are precipitated using cold acetone and resuspended and stored in chloroform/methanol. Following dessication, hydration using water or phosphate-buffered saline with the antigen(s) to be encapsulated followed by size reduction results in multilamellar archaeosomes in the size range of 100–150 nm. These archaeosomes are very stable when stored in suspension [73]. 

Some archaeobacterial-like lipids can be used as cationic lipids or co-lipids for *in vitro* gene transfection [74]. 

### 4.5. Sphingosome

Since liposome stability problems are of course much more severe which led the formation of sphingosomes. Phospholipids used in liposomes are prone to undergo chemical degradation such as oxidation and hydrolysis either as a result of these changes or otherwise liposome maintained in aqueous suspension which may aggregate, fuse, or leak their content. Hydrolysis of ester linkage is responsible for occurrence of this problem. The hydrolysis may be avoided altogether by use of lipid which contains ether or amide linkage instead of ester linkage. Thus sphingolipids are been nowadays used for the preparation of stable liposomes known as sphingosomes. Sphingosome may be defined as “concentric, bilayered vesicle in which an aqueous volume is entirely enclosed by a membranous lipid bilayer mainly composed of natural or synthetic sphingolipid.” 

Liposomal formulation based on sphingomyelin-based cholesterol has several advantages when compared to other formulation. The sphingosomes are much more stable to acid hydrolysis and have better drug retention characteristics. Sphingosomes are administered in many ways which include parentral route of administration such as intravenous, intramuscular, subcutaneous, and intra-arterial. Generally it will be administered intravenous or in some cases by inhalation. Often it will be administered into a large central vein, such as the superior vena cava and inferior vena cava to allow highly concentrated solution to be administered into large volume and flow vessels. Sphingosomes may be administered orally or transdermally [75]. 

### 4.6. Virosomes

Virosomes are reconstituted viral envelopes that composed of a lipid bilayer in which inserted viral glycoproteins can be derived from different enveloped viruses [16]. Virosomes were initially prepared by Almeida et al. [76] and described as liposomes with influenza virus hemagglutinin (HA) and neuraminidase (NA) spikes on their surface. Virosomes closely mimic the intact virus except that they do not contain virus replication machineries. They retain the cell entry and membrane fusion characteristics of the virus they are derived from. There are two pathways reported by which reconstituted vesicles are able to enter cells and deliver their contents into the cytoplasm: plasma membrane fusion (Sendai virus) and acid-induced fusion from within endosomes (Influenza virus). Solubilization and reconstitution are two physical processes which may cause inactivation of membrane proteins. 1,2-dihexanoylphosphatidylcholine (DHPC) is used as a viral membrane solubilizer [77]. The mildly acidic pH in the endosome triggers the exposure of the fusion peptide of the viral HA. This results in fusion of the viral membrane with the endosomal membrane and thereby release of the genome of the virus into the cytoplasm of the cell. As a consequence, foreign substances encapsulated within the lumen of virosomes are effectively delivered to the cytosol of target cells [78]. Virosomes can be used in vaccination, for efficient induction of antibody responses against the virus they are derived from. For example, there is an elegant carrier system for prophylactic and/or therapeutic vaccines in humans against Hepatitis C Virus and other targets [79] and for protection against HIV-1 infection [80].

### 4.7. Escheriosomes

These are lipoidal vesicles, prepared from polar lipids extracted from *Escherichia coli*. Majorly phosphatidyl ethanolamine, cardiolipin, and phosphatidyl glycerol are classes of phospholipid, present in *Escherichia coli * [81]. *E. coli* contains an altered fatty acid and phospholipid composition when grown in the presence of sublethal concentrations of a variety of organic solvents and food additives [82]. But during progression from exponential growth phase to the stationary growth phase, the phospholipid composition of the cell was altered. Unsaturated fatty acids were converted to cyclopropane fatty acids, and phosphatidyl glycerol appears to have been converted to cardiolipin [83]. Also, ethanol was found to decrease the level of lipids in *E. coli * [84]. These novel fusogenic liposomes have strong tendency to fuse with the plasma membrane of target cells and thereby delivering the entrapped contents into their cytosol [85]. Escheriosomes are helpful in controlling intracellular pathogens by expression of particular enzymes [86]. Escheriosomes-encapsulated antigen elicited strong humoral immune response in immunized animals but, in general, escheriosomes are considered as potential candidate vaccine carrier system capable of eliciting both cell-mediated as well as humoral immune responses [87].

Escheriosome-based delivery helps for generating protective immunity against *C. albicans* infection [88], to induce protective immune responses against experimental murine brucellosis [89], developing vaccine against leishmaniasis as well as other intracellular infections [90]. These are emerged as promising delivery vehicle which synergizes the effect of leptospira vaccines [91].

### 4.8. Leptosomes

These are novel type of vesicles prepared from total polar lipids of nonpathogenic *Leptospira biflexa.* These were prepared by Faisal et al. and they evaluated their vaccine delivery/adjuvant potential with novel protective antigens (Lp0607, Lp1118 and Lp1454) of *L. interrogans* serovar Pomona in a hamster model. The immune response induced by three individual antigens and protective efficacy were evaluated and compared to those induced by same antigens entrapped with PC-liposomes and *E. coli* lipid liposomes (escheriosomes). Finally, they concluded that leptosome is better adjuvant than PC-liposomes as revealed by enhanced long-term antibody response, lymphocyte proliferation and significant enhancement of both Th1 (IFN-gamma) and Th2 (IL-4 and IL-10) cytokines [91]. 

### 4.9. Subtilosomes

Fusogenic properties of the liposomes (subtilosome), prepared from phospholipids isolated from *Bacillus subtilis*, make them novel potential carrier system in therapeutics. Cardiolipin and phosphatidyl glycerol are abundant in *B. subtilis*. The transport of various metabolically important substances seems to be possible with these types of vesicles [92].

### 4.10. Lipospheres

One of the most promising systems that has evolved for sustained drug release is the liposome-based delivery system. In this system, the drug is entrapped into lipid bilayers or aqueous compartments of the liposome. However, this system suffers from the disadvantages of high production costs and inherent instability. A novel encapsulation technology referred to as the Liposphere drug delivery system has recently been developed. This system consists of water-dispersible microparticles called lipospheres, each composed of a solid hydrophobic core of triglycerides containing the drug and phospholipids embedded on the surface of the core. The phospholipids provide the necessary means of dispersing the lipospheres in a pharmaceutically acceptable vehicle. Also, this method is simple, rapid, inexpensive, and reproducible [93]. The advantages offered by the liposphere delivery system include ease of manufacture, low production costs of components, high stability of the drug and formulation, and ease of controlling drug release rate by simply manipulating the ratio of triglyceride to phospholipid. The average particle size can also be controlled from a few hundred nanometers to microns [94]. They possessed the ability to entrap the drug at very high levels and high stability, and to sustain the anti-inflammatory [95, 96], antihypertensive [97], antidiabetic [93], antiglaucoma [98], anesthetic [99], anticancer [100, 101], antibacterial [102] effect of the drug. Lipospheres allow for magnetic force-assisted transfection [103] as well as entrapment of enzymes [104].

### 4.11. Ufasomes

Unsaturated fatty acid vesicles (ufasomes) are the suspensions of closed lipid bilayers that are generated at specific pH. The formation of ufasomes is believed to occur due to associative interaction in mixtures of fully ionized and unionized fatty acids at pH>7.0. In ufasomes, fatty acid molecules are oriented in such a way that their hydrocarbon tails are directed toward the membrane interior and the carboxyl groups are in contact with water. Oleic and linoleic acids were used mostly [105]. In biological membranes, the arrangement of lipid molecules exhibits dual role, that is, structural and functional: structural part as they provide matrix for membrane proteins and a functional one, in which they act as a barrier to the free flow of solutes [106]. Stability of ufasomes depends on proper selection of fatty acid, amount of cholesterol, buffer, pH range, amount of lipoxygenase, and the presence of divalent cations. Recent innovations can provide opportunity to formulate ufasomes with tailorable features such as extension of pH range, insensitivity toward divalent cations, and enhancement of stability [107]. But, ufasomes have a less regular structure than conventional liposomes [108]. Ufasomes have potential as carriers for the oral administration of poorly absorbable drugs [109, 110] as well as for the horizontal transfer of genes from plants [111].

### 4.12. Cryptosomes

Cryptosomes are superior type of lipid vesicles with a surface coat results from self-assembly of suitable polyoxyethylene (PEG) derivatives of phosphatidylethanolamine. The name originated from the greek words “cryptos”: hidden and “soma”: body. The life-time and the distribution of the stabilized lipid vesicles were found to be more than standard liposomes made of phosphatidylcholine only, as former circulate 8–10 times more in blood stream. Mixture of distearoylphosphatidylethanolamine-PEG (DSPE-PEG) with distearoylphosphatidylcholine can employed for cryptosome formation [112, 113]. These long circulating liposomes reduce mononuclear phagocyte system uptake [114]. High-phase-transition temperature phospholipids, a high fraction of cholesterol, and a small fraction of some specific glycolipids (e.g., monosialoganglioside and hydrogenated phosphatidylinositol) imparting a weak surface negative charge were recognized as factors contributing to the longer circulation half-lives of liposomes [115]. Cryptosomes were found to be superior to non-stealth liposomes in prolonging mean survival times in animal models [116].

### 4.13. Emulsomes

The emulsome nanocarrier technology is a lipid-based drug delivery system designed to act as a vehicle for drugs with poor water solubility. As one kind of new drug carrier, emulsomes were studied more in recent years; it is one kind of new dosage form belonging to target-oriented and sustained drug delivery system [117]. Emulsomes are a new generation of colloidal carrier systems in which internal core is made of fats and triglycerides which is stabilized by high concentration of lecithin in the form of o/w emulsion. Emulsomes itself intact have the characteristics of both liposomes and emulsions. By virtue of solidified or semisolidified internal oily core, it provides a better opportunity to load lipophilic drugs in high concentration, simultaneously a protracted controlled release can also be expected and the ability to encapsulate water-soluble medicaments in the aqueous compartments of surrounding phospholipids layers [118].

The solvent-free and surfactant-free emulsome technology has demonstrated high drug-encapsulation capacity for water-insoluble antifungal [119] and anticancer drugs showing enhanced drug delivery and improved preclinical efficacy for parenteral routes. An example of the successful application of the technology is the development of an injectable ready-to-use emulsome-based formulation for the antifungal agent amphotericin B [120].

Advantages:

- superior bioavailability,
- reduced toxicity,
- improved pharmacological activity [121].

### 4.14. Marinosomes

Marinosomes are liposomes based on a natural marine lipid extract containing high ratio polyunsaturated fatty acids like eicosapentaenoic acid (EPA) and docosahexaenoic acid (DHA). They are not present in normal skin epidermis [122]. However, they are metabolized by skin epidermal enzymes into anti-inflammatory and antiproliferative metabolites that are associated with a variety of benefits with respect to inflammatory skin disorders by regulating PGE2 and IL-8 production in human keratinocyte cultures. However, the preventing effect of Marinosomes was highly dependent on the lipid concentration used and the liposome mean diameter [123]. Active and passive loading of drug, as well as complex structural rearrangements [124], directly depends on transmembrane pH gradient [125]. All these results allowed considering Marinosomes as potential candidates for cosmeceutical and oral PUFA supplements in view of the prevention and treatment of deficiencies [126–128].

### 4.15. Enzymosomes

Different strategies can be used to improve carrier-mediated delivery of therapeutic proteins. The incorporation of therapeutic enzymes into polymeric carriers into aqueous space of lipid vesicles or lipid-detergent vesicles and incorporation of hydrophobized forms into lipid bilayer of vesicles are strategies to be used. None of above mentioned can save the therapeutic protein completely. Another strategy, not usually used for therapeutic enzymes, is its attachment to the outer surface of liposomes, using technologies developed for antibodies. Enzymes on complex with lipids generate enzymosome. Preservation of enzyme activity and preservation of vesicles structural integrity were two desirable features from enzymosome [129, 130]. *In vitro* antitumor activity experiments showed that the improved immunoenzymosome system is able to completely convert the prodrug daunorubicin-glucuronide into its parent compound [131]. They have considerably improved enzyme targeting capability [132].

### 4.16. Genosomes

A genosome is complex of genetic material like DNA and suitable lipid. They are also known as Lipoplexes that are used to deliver genes. Mostly DNA-cationic liposome complexes were used to translocate DNA across cellular membranes *in vivo*, because interaction between DNA-lipid membranes has proved crucial to the understanding of the colloidal state of the genosomes. These DNA-lipid complexes could be later aggregated into higher order assemblies, creating stacked lipid-DNA multilayers, for generating more protection [133, 134]. The interesting features of the strong ordering of DNA as well as lipid bilayers in a genosome are explained in terms of colloidal forces and strong DNA-lipid interactions [135].

### 4.17. Miscellaneous

Vesicular system approaches possess vital role to deliver a drug by different route to achieve better therapeutic action. To resolve various drawbacks, researchers are implementing their efforts in improving the design of vesicular system. Some more lipoidal vesicular carriers are highlighted below like erythrosomes [136], vesosomes [137], cubosomes [138], and hexosomes [139].

## 5. Nonlipoidal Biocarriers

This paper mainly focused on lipoidal soft hybrid structures, but we enlightened some non-lipoidal vesicles to present a comparative view between them. Nonlipoidal drug delivery has been studied using various methods of administration including intramuscular, intravenous, peroral, and transdermal. Non-lipoidal vesicles have equal potential of drug delivery of various bioactives. These include the following. 

### 5.1. Niosomes

The greater stability, ease of preparation, and economical aspects of nonionic surfactant have led to exploitation of these compounds as alternatives to phospholipids. In recent years, niosomes received great attention as potential drug delivery systems for different routes of administration. Niosomes are microscopic lamellar structures formed on admixture of a nonionic surfactant, cholesterol and charge inducers with subsequent hydration in aqueous media. This system was found to accommodate both hydrophobic and hydrophilic drug either in aqueous or bilayer region of vesicles [140, 141]. 

Nonionic surfactant vesicles (NSVs or niosomes) result from the self-assembly of hydrated surfactant monomers. They are similar in physical structure and form to the more widely studied phospholipid vesicles (liposomes). The association of nonionic surfactant monomers into vesicles on hydration is a result of the fact that there exists a high interfacial tension between water and the hydrocarbon portion (or any other hydrophobic group) of the amphiphile which causes these groups to associate. Simultaneously, the steric, hydrophilic, and/or ionic repulsion between the head groups ensures that these groups are in contact with water. These two opposing forces result in a supramolecular assembly. Sterically stabilized, emulsified, polymerized nonionic surfactant systems were found to be modified nonionic surfactant systems [142]. 

The cost-effectiveness and variable purity of non-ionic surfactant militate to choose niosomes as drug delivery vesicles and made them potential carrier system in treatment of tuberculosis [143], glaucoma [144], skinredness,irritation,itching [145], fungal infections [146, 147], osteoarthritis, rheumatoid arthritis, and spondylitis [148].

### 5.2. Proniosomes

Proniosomes are dry formulation of solid colloidal particles that are coated with surfactant and can be converted into niosomes by agitation in water for a short time. These water-soluble carrier particles are very similar to conventional niosomes and more uniform in size [149]. It presents a useful vesicle-delivery concept with potential to deliver drugs via the transdermal route. This would be possible if proniosomes form niosomes upon hydration with water from skin following topical application under occlusive conditions [150]. These can be formulated either by spraying method or slurry method [151]. Proniosomes minimize problems of niosomes physical stability such as aggregation, fusion, and leaking and provide additional convenience in transportation and dosing [152]. The proniosome approach minimizes these problems by using dry, free-flowing product, which is more stable during sterilization and storage. Ease of transfer, distribution, measuring, and storage make proniosomes a versatile delivery system with potential for use with a wide range of active compounds [153].

Maltodextrin-based proniosomes were found to be a potentially scalable method for producing niosomes for delivery of hydrophobic or amphiphilic drugs [154]. For the delivery of variety of nonsteroidal anti-inflammatory and analgesics, proniosomes possess a remarkable potential [155–157]. 

### 5.3. Bilosomes

Bilosomes as recent innovative drug delivery carriers consist of deoxycholic acid incorporated into the membrane of niosomes. Optimum blend of bile salts in niosomal formulation could stabilize the membrane against the detrimental effects of bile acids in GI tract. These bile salt stabilized vesicles referred to as “Bilosomes.” Additionally, Bile salts are commonly used as penetration enhancers. Bilosomes endow a list of advantages including biocompatibility as they are produced from naturally occurring lipids. Bile salts along with lipid content increase the bioavailability of enclosed bioactive. Also, this delivery system exhibits inherent adjuvant properties when associated with antigen. These allow small quantities of antigen to be effective and both cellular and humoral immune responses can be induced [158, 159].

Shukla et al. showed that HBsAg loaded bilosomes produced both systemic as well as mucosal antibody responses upon oral administration [160]. Furthermore, bilosomes with a five times higher dose upon oral administration produced comparable serum antibody titres to those obtained after intramuscular immunization without the induction of systemic tolerance. This approach solved two major problems associated with available marketed hepatitis B vaccines administered through parenteral route, primarily, degradation of antigen in the harsh and hostile environment of the gastrointestinal tract and inability to induce a mucosal antibody response [160].

Bilosomes have profound implications for future vaccine development and indicate the potential for increasing the immunization success rate. In studies of Mann et al. [158], they proved that bilosome entrapped influenza HA not only induces significant-specific systemic antibody production but also mucosal IgA. This has not previously been demonstrated [158].

For extended humoral, cell-mediated and mucosal immune responses, additional coating carrier system was found better protecting against disease for prolonged period of time. Optimum mannan coating was found to stabilize the vesicles in gastrointestinal environment as well as act as a targeting ligand for mannose receptors expressed on macrophages and dendritic cells [159]. 

### 5.4. Aspasomes

Ascorbyl palmitate in presence of cholesterol and charge inducer dicetyl phosphate encapsulate drug in its core to form aspasomes. The antioxidant potency of aspasome was much better than that of ascorbic acid [161, 162]. Thus, it can find applications as drug delivery system in disorders implicated with reactive oxygen species. Aspasomes enhanced the transdermal permeation of azidothymidine. The antioxidant property and skin permeation enhancing property indicate a promising future for aspasome as a carrier for transdermal drug delivery system [161, 163].

### 5.5. Miscellaneous

These systems are preferred over other vesicular systems as they offer advantages such as being biodegradable, biocompatible and non-immunogenic, osmotically active and stable. Aquasomes [164], polymersomes [165], colloidosomes [166] are novel promising nonlipoidal drug carriers and have potential to reduce the side effects of drugs as well as increase therapeutic effectiveness in various diseases.

## 6. Conclusion

The application of vesicular system in drug delivery has changed the definitions of diagnosis and treatment in different aspects of biomedical field. The concept of incorporating the drug into vesicles for a better targeting of the drug at appropriate tissue destination is widely accepted. It is obvious that various deformable as well as rigid, lipid supramolecular constructed vesicles have great drug delivery potential for targeted delivery of various bioactives. In the last couple of years, continuous research have been going on for better delivery of drugs with the aim of better targeting and minimization of dosing frequency. With the concepts of supramolecular chemistry, novel vesicular systems are capable to acquire-above-mentioned qualities. To fulfill these tasks, these may be formulated into liquid or semi-liquid drug delivery systems with phospholipids. These novel vesicular systems showed their therapeutic potential from topical to genetic levels. Thus, it appears that supramolecular vesicular delivery systems will continue to thrive as a useful tool in pharmaceutics for the improvement of drug solubility, oral absorption, and hence bioavailability. 

## Figures and Tables

**Table 1:** Comparison of few aspects of lipoidal particulate carriers and their applications.

S. no.	Carrier	Composition	Entrapped agent	Unique features	References
1	Liposomes	Phospholipids:cholesterol:alcohol	Antibiotics, antineoplastic agents, antitubercular drugs	Amphiphilic nature provides solubilization of both hydrophilic and lipophilic drugs, internalisation and amplification of bioactives	[20, 21]
2	Transfersomes	Phospholipids:edge activators:alcohols:buffering agent:dye	NSAIDs, anesthetics, steroidal hormones	Ultradeformable vesicles can deform and pass through narrow constriction (from 5 to 10 times less than their own diameter) without measurable loss	[22–30]
3	Pharmacosomes	Phospholipids:dichloromethane	NSAIDs	Colloidal dispersions of drugs covalently bound to lipids, which increased entrapment efficiency; no loss of drug due to leakage, no problem of drug incorporation	[31–33]
4	Ethosomes	Phospholipids:ethanol	Antifungal agent, antiviral agent, antikeratinizing agent, NSAIDs	Combinational approach of high concentration of ethanol along with phospholipids synergizes effect of deeper distribution and penetration of drugs in the skin	[17, 34–42]

**Table 2:** Provesicular candidates and their features.

S. no.	Carrier	Preparation procedure	Unique features	Therapeutic indication	References
1	Proliposomes	Dehydration-rehydration method	Dry free-flowing powder can be hydrated immediately to form liposomes through contact with water or biological fluids	Tuberculosis	[47–51]
2	Protransfersomes	Hand shaking method	Proultraflexible lipid vesicles (protransfersomes) would be converted into ultraflexible vesicles *in situ* by absorbing water from the skin	Cutaneous squamous cell carcinoma	[52]
